# Effects of Partial-Body Cryotherapy on Athletic Performance and Sleep Quality in Division I Collegiate Basketball Athletes

**DOI:** 10.5114/jhk/203236

**Published:** 2025-04-30

**Authors:** Tae-Jin Kim, Kyeong-Hyon Ha, Tae-Young Park, Jung-Hyun Kim, Jung-Min Lee, Hyun Chul Jung

**Affiliations:** 1 Department of Physical Education, Graduate School, Kyung Hee University, Yongin, Republic of Korea.; 2 Department of Sports Medicine, Kyung Hee University, Yongin, Republic of Korea; 3 Sports Science Research Center, Kyung Hee University, Yongin, Republic of Korea.; 4 Department of Physical Education, Kyung Hee University, Yongin, Republic of Korea.; 5 Department of Sports Coaching, Kyung Hee University, Yongin, Republic of Korea.

**Keywords:** recovery, cryostimulation, physical performance, ergogenic aids, sleep deprivation

## Abstract

This study examined the effects of short-term partial-body cryotherapy (PBC) on athletic performance and sleep quality in Division I collegiate basketball athletes. A crossover, counter-balanced design was employed with twelve collegiate basketball athletes randomly assigned to five days of a post-exercise PBC condition or a control condition. Athletic performance was assessed using six standardized tests from the Korean Basketball League (KBL) Draft combine. Objective and subjective sleep quality were measured using actigraphy and three validated sleep questionnaires, respectively. The number of pull-ups performed significantly increased after the PBC condition (9.2 ± 4.59 vs. 11.9 ± 4.77 reps, p < 0.05), whereas no improvements were observed in other performance measures, including the maximum repetitions of the 75-kg bench press, sprint speed, vertical jump height, and agility. Subjective and objective sleep quality were not enhanced after the PBC condition. These findings suggest that a short-term (5-day) application of PBC has only a limited effect on athletic performance and no effect on sleep quality. Future studies with longer intervention periods are needed to better understand the effects of PBC on athletic performance and sleep quality.

## Introduction

Overload is an essential part of training to improve performance, but excessive training stress and insufficient recovery can lead to severe fatigue and chronic maladaptation, affecting metabolic, immune, and hormonal function (Cadegiani and Kater, 2017; Meeusen et al., 2013; Purvis et al., 2010). Studies show that 20–60% of athletes experience the negative effects of overtraining at least once in their careers (Nederhof et al., 2006).

The attainment of optimal recovery is crucial for sustaining peak performance levels during repetitive training and competition (Halson, 2013). Sleep plays a vital role in this recovery process, as inadequate sleep is linked to overtraining, reduced performance, and impaired muscle protein synthesis (Fullagar et al., 2015; Lastella et al., 2018). However, studies indicate that 71% of elite athletes experience insufficient sleep, with further reductions in sleep quality and quantity occurring during periods of high training loads (Lastella et al., 2018; Sargent et al., 2021). In-season athletes not only endure successive games and training sessions, but also face off-court challenges such as extensive travel, which can limit opportunities for sufficient rest and recovery (Davis et al., 2022). Consequently, implementing strategies to improve sleep quality is critical for elite athletes to optimize recovery within limited recovery windows.

Partial-body cryotherapy (PBC) using a cryochamber has gained attention as a potential strategy that may facilitate post-exercise recovery. PBC involves brief exposure (2–3 minutes) to extremely low temperatures (−100 to −195°C) in a controlled chamber environment (Ferreira-Junior et al., 2014). This exposure reduces tissue temperature, leading to physiological effects such as decreased skin microcirculation, muscle metabolism, receptor sensitivity, and neural conductance velocity (Costello et al., 2015). These mechanisms contribute to reducing muscle soreness and damage, as evidenced by findings showing PBC-induced reductions in post-exercise creatine kinase activity and increases in anti-inflammatory cytokines (Costello et al., 2015; Wozniak et al., 2007). Despite evidence indicating that cryostimulation helps alleviate muscle pain and damage (Trybulski et al., 2024), its impact on functional recovery remains inconclusive (Bleakley et al., 2014).

Moreover, PBC has been proposed as a strategy to enhance sleep quality, possibly due to its analgesic properties (Douzi et al., 2019a). Research has suggested that post-exercise cryostimulation may also facilitate reactivation of the parasympathetic nervous system, a physiological response that could improve sleep quality (Douzi et al., 2019a). While some studies have reported improvements in sleep variables following PBC exposure (Bouzigon et al., 2014; Schaal et al., 2015), existing evidence remains limited and largely reliant on subjective assessments. Given that self-reported sleep duration by male collegiate athletes is often overestimated (Carter et al., 2020), it is essential to incorporate objective sleep variables, such as actigraphy, alongside subjective measures from questionnaires and scales to provide a comprehensive evaluation of PBC’s effects on sleep quality.

Therefore, this study aimed to examine the effects of a 5-day PBC on 1) athletic performance and 2) sleep quality in in-season elite athletes.

## Methods

### Participants

Participants’ characteristics are presented in Table 1. Fourteen male collegiate basketball athletes, who were registered as Division I collegiate athletes in the Korean Basketball Association, were recruited for the study. According to the classification framework proposed by McKay et al. (2022), participants in this study fell into tier 3. The inclusion criteria for this study were as follows: 1) participants aged 18–30 years, 2) able to participate in athletic performance tests. Participants were excluded if they had a cold-related illness, claustrophobia, a history of acute respiratory or cardiovascular disease or a musculoskeletal injury that limited their participation in training. This study was approved by the Institutional Review Board of the Kyung Hee University, Yongin, Republic of Korea (protocol code: KHUIRB-22-323; approval date: 02 August 2022) and complied with the principles of the Declaration of Helsinki. All participants received information about the study procedures, purpose, and potential risks of the experiment before providing written consent. During the study period, two athletes did not complete the study due to injury or personal reasons; thus, data from 12 participants were analyzed.

**Table 1:** Participants' characteristics.

Variables	Mean ± SD
Age (yrs)	20.8 ± 1.22
Body height (cm)	187.1 ± 6.12
Body mass (kg)	80.8 ± 6.87
BMI (kg•m^−2^)	23.1 ± 1.21
% Body fat	14.4 ± 2.45
Lean mass (kg)	69.2 ± 5.91
Fat mass (kg)	11.7 ± 2.34
Training experience (yrs)	8.2 ± 2.95

### Measures

#### Anthropometry and Body Composition

Body height and mass were measured to the nearest 0.1 cm and 0.1 kg, respectively, with a stadiometer (Aluminum anthropometer, Samhwa instruments, South Korea, 1996) and a digital weighing scale (CAS 150A, Seoul, South Korea, 1999). DXA (QDR-4500 Elite, Hologic, MA, USA, 2004) was used to assess body composition including the body fat percentage, lean body mass, and fat mass.

#### Athletic Performance

Athletic performance was assessed following the Korean Basketball League (KBL) draft combine procedure. The procedure consisted of six tests, which were performed in the following sequence: a maximum repetition bench press test, a maximum repetition pull-up test, a vertical jump test, a 10-yard sprint, a 3/4 court (22.86 m) sprint, and a lane agility test. These tests were selected because they are part of the procedures used to evaluate the fundamental athletic performance of rookie players in the professional basketball league that our participants aimed to join after graduation. The bench press and pull-up tests were performed once. A weight of 75 kg was set for the maximum repetition bench press test. Pull-ups were performed with participants’ body weight. The vertical jump, the 10-yard sprint, the 3/4 court sprint, and the lane agility drill were each performed twice. Higher values were registered for further analysis for the vertical jump, while lower values were considered for the 10-yard sprint, the 3/4 court sprint, and the lane agility drill. The vertical jump was performed on a timing mat (Just Jump System, Probotics, Huntsville, AL, USA) with hands placed on the waist. The 10-yard sprint, 3/4 court sprint, and lane agility drill times were measured using a stopwatch (HS-3, Casio, Tokyo, Japan). The 10-yard sprint time was recorded during the 3/4 court sprint to minimize fatigue. The bench presses and pull-ups were recorded as the number of repetitions, the vertical jump height in centimeters (cm), and the 10-yard sprint, 3/4 court sprint, and lane agility times in seconds (s).

To evaluate the test-retest reliability of performance measures, intraclass correlation coefficients (ICCs) were calculated. The ICCs were computed using a two-way mixed-effects model with absolute agreement to determine the consistency of measurements between the two sessions. All performance measures demonstrated excellent test-retest reliability: 0.97 for bench presses, 0.97 for pull-ups, 0.98 for vertical jumps, 0.90 for the 10-yard sprint, 0.95 for the 3/4 court sprint, and 0.93 for the lane agility test.

#### Sleep Quality

Subjective sleep quality was assessed every day during the treatment period, and the average value was used for further analysis. The Korean versions of the Pittsburgh Sleep Quality Index (PSQI-K), the Insomnia Severity Index (ISI-K), and the Epworth Sleepiness Scale (ESS-K) were used to assess subjective sleep quality. The translated versions of the PSQI, the ISI, and the ESS were validated by Sohn et al. (2012), Cho et al. (2014), and Cho et al. (2011), respectively. Additionally, 1 to 10 numeric rating scales for sleep (NRS-Sleep) and visual analog scales for perceived pain (VAS) data were gathered during the treatment period.

Objective sleep quality was assessed using an accelerometer (Actigraph, wGT3X-BT, USA) and analyzed with ActiLife software version 6.11.0 (Fort Walton Beach, FL, USA). The accelerometer used in the present study showed excellent agreement, low mean bias, and narrow limits of agreement for all sleep variables when compared to another actigraphy device (Buchan, 2024). The Actigraph captured movements on the horizontal, vertical, and perpendicular axes and the epoch length was set to 60 s. Data were collected every day during the treatment period and the average value of three consecutive days (3^rd^–5^th^ visit, 10^th^–12^th^ visit) was used for analysis. Objective sleep variables included total time in bed, total sleep time, wake after sleep onset, and sleep efficiency.

#### Partial-Body Cryotherapy

Participants underwent five sessions of PBC exposure using a cryocabin (KRION standard, KRION, Russia). PBC was conducted 10 minutes after the completion of the training session. The timing (18:00–19:00) of PBC was matched throughout the treatment period. The temperature of the PBC cabin was lowered by liquid nitrogen injection. Participants began the PBC session when the cabin temperature reached at least −110°C, gradually decreasing to −170 to −196°C by the end of the exposure. Feet and hands were protected with warm shoes and gloves, while participants’ heads remained outside the cabin. Each PBC session lasted up to three minutes, with participants being asked every 30 s whether they could continue the exposure. The mean and standard deviation of the exposure time was 127.7 ± 40.06 s.

### Design and Procedures

**Figure 1:**
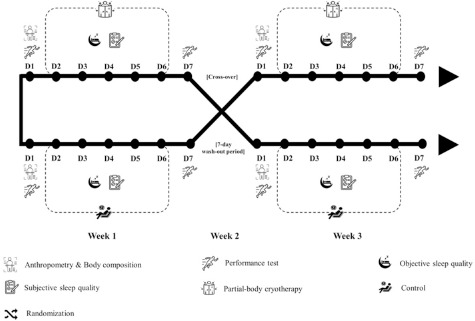
The schematic view of the study design.

The present study was conducted using a crossover and counter-balanced design. The schematic view of the study design is presented in Figure 1. On the 1^st^ visit, participants’ height, body mass, body composition, and athletic performance were assessed. Participants underwent a 24-hour washout period before commencing the treatment. During the 5-day treatment period (2^nd^–6^th^ visits), participants were allocated to either a control or a PBC condition. Participants followed the same training and competition schedules during both conditions. Under the control condition, participants did not receive any post-exercise recovery treatments and were instructed to maintain their habitual lifestyle. Both subjective and objective sleep quality data were collected throughout the 5-day treatment period. After the 6^th^ visit, participants had another 24-hour washout period. On the 7^th^ visit, post-measurements of participants’ athletic performance were conducted. After the 7-day washout period, participants switched to the alternative condition and underwent the same procedure as in the 1^st^–7^th^ visit.

During the experimental period, participants took part in training according to the following schedule. On non-match days, weight training and individual skill training were conducted during the morning and evening sessions. Additionally, team tactical training was carried out during the afternoon session, resulting in a total of three training sessions per day. On match days, team tactical training was held in the morning, followed by the official match in the afternoon.

### Statistical Analysis

All data were analyzed using the IBM SPSS Statistics version 28.0 software package for Windows (SPSS Inc., Chicago, IL, USA). The Shapiro-Wilk test was used to determine the normality of data distribution. This study employed a crossover design with a within-subject (2 × 2 factorial) design, where factor 1 was condition and factor 2 was time. A linear mixed model was used to analyze the effect of PBC, accounting for missing values in the performance data. Time was converted into a categorical variable, and condition, time, and the condition × time interaction were set as fixed effects, while subjects were treated as random effects. When a significant interaction effect or main effects were found, post-hoc tests were conducted using pairwise comparisons with Bonferroni correction. Quantitative sleep quality was assessed using the average of data measured over three days (Monday, Tuesday, and Wednesday) during the PBC and control periods. The dependent-sample *t*-test was used to compare differences in means. The questionnaire data were analyzed using a two-way (2 × 2) ANOVA with repeated measures. When a significant interaction effect or main effects were found, post-hoc tests were conducted using pairwise comparisons with Bonferroni correction. The significance level (α) was set at 0.05. The sample size was determined using G*power version 3.1 based on pilot data from our laboratory. Based on an effect size of 0.4, an alpha risk of 0.05, and a desired power of 0.8, the results indicated that 12 participants were needed.

## Results

**Figure 2:**
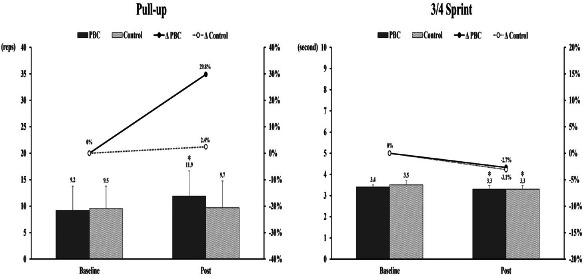
Changes in the pull-up test and the 3/4 court sprint after intervention periods. *: significant differences from baseline

The effects of PBC on athletic performance are presented in Tables 2 and 3, and Figure 2. For the 75-kg bench press, a significant time effect was found (*F* = 4.69, *p* < 0.05), but the coefficient was not significant (*t* = −1.88, *p* = 0.08). There was no significant difference in the number of 75-kg bench press repetitions performed between the baseline and post-test. A significant interaction effect was found in pull-ups (*F* = 8.01, *p* = 0.01). The number of pull-ups performed significantly increased after the PBC condition (9.2 ± 4.59 vs. 11.9 ± 4.77 reps, *p* < 0.01), whereas no significant change was observed in the control condition. A significant time effect was found for the 3/4 court sprint (*F* = 10.56, *p* < 0.01). The 3/4 court sprint time significantly decreased after both the control (−3.1%, *p* = 0.02) and the PBC condition (−2.7%, *p* = 0.05). For the Sargent jump, the 10-yard sprint, and lane agility, there were no significant interaction effects or main effects.

**Table 2:** Results of linear mixed models on athletic performance.

Variables	Model summary		Parameter estimates					
	*F*-value	*p*-value	Coefficient	*SE*	*t*-value	*p*-value	95% CI	Cohen’s *f*^2^
**75-kg bench press (reps)**								
Condition	< 0.01	0.97	−0.26	1.60	−0.16	0.87	[−3.57, 3.05]	0.02
Time	4.69	< 0.05	−1.20	0.64	−1.88	0.08	[−2.54. 0.14]	0.04
Interaction	0.19	0.67	0.40	0.92	0.44	0.67	[−1.54, 2.35]	0.01
**Pull-ups (reps)**								
Condition	0.09	0.77	−1.47	1.94	−0.76	0.46	[−5.48, 2.53]	0.05
Time	25.33	< 0.01	−2.51	0.46	−5.44	< 0.01	[−3.48, −1.54]	0.10
Interaction	8.01	0.01	1.81	0.64	2.83	0.01	[0.47, 3.14]	0.04
**Sargent jump (cm)**								
Condition	0.02	0.88	1.09	2.00	0.55	0.59	[−3.00, 5.19]	0.04
Time	2.20	0.15	2.51	1.10	2.29	0.03	[0.23, 4.79]	0.04
Interaction	3.29	0.08	−2.76	1.52	−1.81	0.08	[−5.92, 0.40]	0.02
**10-yard sprint (s)**								
Condition	4.02	0.06	−0.06	0.05	−1.21	0.23	[−0.15, 0.04]	0.08
Time	1.29	0.27	0.05	0.04	1.14	0.27	[−0.04, 0.14]	0.13
Interaction	0.25	0.62	−0.03	0.06	−0.50	0.62	[−0.16, 0.09]	0.06
**3/4 court sprint (s)**								
Condition	0.32	0.58	0.03	0.07	0.39	0.70	[−0.12, 0.17]	0.23
Time	10.56	< 0.01	0.09	0.04	2.08	0.05	[0.00, 0.18]	0.02
Interaction	0.07	0.80	0.02	0.06	0.26	0.80	[−0.11, 0.14]	0.12
**Lane agility (s)**								
Condition	0.06	0.81	−0.14	0.18	−0.77	0.45	[−0.51, 0.23]	0.04
Time	0.17	0.69	−0.12	0.09	−1.41	0.17	[−0.30, 0.06]	0.09
Interaction	2.52	0.13	0.19	0.12	1.59	0.13	[−0.06, 0.45]	< 0.01

SE: standard error, 95% CI: 95% confidence interval for coefficient

**Table 3:** Results of pairwise comparison with Bonferroni correction for linear mixed models.

Variables	Intervention	Time		% Δ	ICC
		Baseline	Post		
75-kg bench press (reps)	PBC	5.4 ± 3.31	6.6 ± 3.34	22.2%	0.97
	Control	5.6 ± 3.04	6.4 ± 4.77	16.0%	
Pull-ups (reps)	PBC	9.2 ± 4.59	11.9 ± 4.77*	29.8%	0.97
	Control	9.5 ± 4.30	9.7 ± 5.06	2.4%	
Sargent jump (cm)	PBC	51.3 ± 5.71	48.9 ± 4.85	−4.7%	0.98
	Control	49.7 ± 4.91	49.9 ± 3.90	0.4%	
10-yard sprint (s)	PBC	1.9 ± 0.10	1.8 ± 0.11	−2.6%	0.90
	Control	1.8 ± 0.13	1.8 ± 0.12	−1.1%	
3/4 court sprint (s)	PBC	3.4 ± 0.10	3.3 ± 0.18*	−2.7%	0.95
	Control	3.5 ± 0.21	3.3 ± 0.17*	−3.1%	
Lane agility (s)	PBC	11.5 ± 0.36	11.6 ± 0.42	0.9%	0.93
	Control	11.6 ± 0.37	11.5 ± 0.51	−0.4%	

Data are presented as mean ± standard deviation.

* indicates a significant difference compared with the baseline test, PBC: Partial-body cryotherapy

**Figure 3:**
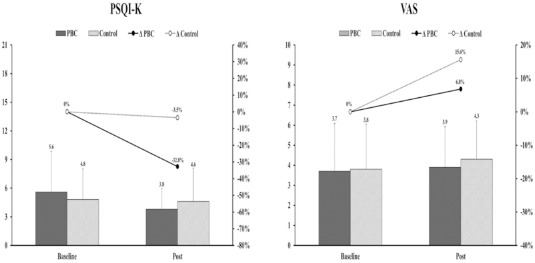
Changes in the PSQI-K and the VAS after intervention periods. PSQI-K: Korean version of the Pittsburgh sleep quality index, VAS: visual analogue scale for perceived pain

The effects of PBC on objective sleep quality are presented in Table 4. There were no significant differences in total time in bed, total sleep time, wake after sleep onset, and sleep efficiency between the PBC and control conditions. The effects of PBC on subjective sleep quality, as measured by questionnaires, and perceived pain, as assessed by the VAS, are presented in Table 5 and Figure 3. In the NRS-sleep, PSQI-K, ISI-K, ESS-K, and VAS, there was no significant interaction effect or main effect. However, although not statistically significant (*F* = 2.97, *p* = 0.11), the PSQI-K showed a 32.8% decrease following PBC with a large effect size (η_p_^2^ = 0.21). Similarly, the VAS showed a large increase after the control condition (15.6%) compared to the PBC condition (6.8%) and exhibited a large effect size (*F* = 3.87, *p* = 0.08, η_p_^2^ = 0.26).

**Table 4:** The effects of partial-body cryotherapy on objective sleep quality.

Variables	Intervention		*t*-value	*p*-value	Cohen's *d*
	PBC	Control			
Total time in bed (min)	461.6 ± 28.03	465.5 ± 20.85	−0.38	0.71	0.11
Total sleep time (min)	366.1 ± 49.06	364.8 ± 35.25	0.12	0.91	0.04
Wake after sleep onset (min)	92.0 ± 29.67	95.6 ± 35.09	−0.87	0.40	0.25
Sleep efficiency (%)	79.0 ± 7.04	78.4 ± 7.24	0.62	0.40	0.18

Data are presented as mean ± standard deviation, PBC: Partial-body cryotherapy

**Table 5:** Results of 2×2 repeated measures ANOVA for subjective sleep quality and perceived pain.

Variables		Time		% Δ		*F*-value (η_p_^2^)	*p*-value
		Baseline	Post				
NRS-Sleep	PBC	5.4 ± 2.39	5.3 ± 2.53	−3.1%	Condition	0.15 (0.01)	0.70
					Time	0.10 (0.01)	0.76
	Control	5.3 ± 2.70	5.1 ± 3.06	−3.2%			
					Interaction	< 0.01 (< 0.01)	1.00
PSQI-K	PBC	5.6 ± 4.25	3.8 ± 2.18	−32.8%	Condition	< 0.01 (< 0.01)	1.00
					Time	2.97 (0.21)	0.11
	Control	4.8 ± 3.22	4.6 ± 3.50	−3.5%			
					Interaction	3.04 (0.22)	0.11
ISI-K	PBC	8.8 ± 5.64	8.6 ± 6.53	−1.9%	Condition	< 0.01 (< 0.01)	1.00
					Time	0.02 (< 0.01)	0.90
	Control	8.4 ± 6.22	8.9 ± 5.79	5.9%			
					Interaction	0.13 (0.01)	0.72
ESS-K	PBC	7.0 ± 5.10	7.5 ± 3.99	7.0%	Condition	0.35 (0.03)	0.56
					Time	1.17 (0.10)	0.30
	Control	6.7 ± 3.87	7.1 ± 3.42	6.2%			
					Interaction	0.02 (< 0.01)	0.90
VAS	PBC	3.7 ± 2.39	3.9 ± 2.02	6.8%	Condition	0.20 (0.02)	0.66
					Time	3.87 (0.26)	0.08
	Control	3.8 ± 2.26	4.3 ± 1.92	15.6%			
					Interaction	0.29 (0.03)	0.60

Data are presented as mean ± standard deviation; NRS-Sleep: 1 to 10 numeric rating scale for sleep, PBC: Partial-body cryotherapy, PSQI-K: Korean version of the Pittsburgh sleep quality index, ISI-K: Korean version of the insomnia severity index, ESS-K: Korean version of the Epworth sleepiness scale, VAS: Visual analogue scale for perceived pain

## Discussion

Despite the rising prominence of PBC as a post-exercise recovery modality, its effects remain controversial (Bouzigon et al., 2021). Therefore, this study aimed to examine the effects of a brief, five-day post-exercise PBC regimen on athletic performance and sleep quality. Two primary observations were made in this study. Firstly, post- workout cryostimulation had a limited impact on improving athletic performance. Specifically, this study assessed the muscular endurance of the upper-body pulling and pushing muscles, vertical jump ability, short-distance sprint performance, and agility. Among these, only the muscular endurance of the pulling muscles showed a significant improvement following the PBC condition. Secondly, over the five-day PBC period, neither subjective nor objective sleep quality showed improvements.

In the present study, a notable increase in upper limb muscular performance (pull-ups) was observed after the PBC condition. This finding aligns with the results of a prior study, which documented substantial enhancements in maximal isometric grip strength within the cryotherapy-treated cohort compared to the control group (De Nardi et al., 2017). It is noteworthy that the reduction in pain can be plausibly attributed to heightened force production by skeletal muscle (Partridge et al., 2019). Although statistical significance was not achieved (*p* = 0.08), it is imperative to note the smaller increase in VAS scores with a large effect size (η_p_^2^ = 0.26), following the PBC condition compared to the control condition. Well-managed pain after cryotherapy may be linked to the observed improvement in muscular endurance.

As per the data presented, it is evident that the application of PBC did not yield a discernible improvement in the objective sleep quality of basketball players. Our findings align with a prior investigation that scrutinized the impact of cryotherapy on sleep quality among elite rugby athletes during their competitive season (Grainger et al., 2019). Notably, post-exercise cryotherapy did not improve sleep quality in that study (Grainger et al., 2019). However, other studies (Douzi et al., 2019a, 2019b) suggest that cryotherapy can serve as a viable method to improve sleep quality under specific conditions. Douzi et al. (2019b) observed a reduction in nocturnal movements after a 3-min continuous cryotherapy exposure. Skin temperature reduction serves as a critical determinant of the effectiveness of PBC. It should be noted that a reduction in nocturnal movements was only observed in the 180-s condition which induced the largest decrease in skin temperature, while the 90-s condition and the 90-s × 2 condition (interspersed by 5-min room temperature rest) did not induce the same degree of skin temperature reduction, resulting in the absence of observed improvements in sleep quality (Douzi et al., 2019b). This dose-dependent effect of cryotherapy assumes significance, because many participants in the present study were unable to complete (127.7 ± 40.06 s) the full 180-s PBC protocol.

Another potential explanation for the lack of improvement in sleep quality following cryotherapy could be related to the timing of its application. Prior studies have demonstrated that passive body heating in the evening enhances sleep quality by facilitating peripheral vasodilation, increasing heat dissipation, and thereby promoting the necessary decline in core body temperature for sleep onset (Haghayegh et al., 2019). Considering that passive body heating, an intervention physiologically opposite to cryostimulation, has shown positive effects when conducted in the evening, our choice to apply cold exposure at this time may have counteracted these beneficial thermoregulatory effects. Therefore, further research is needed to systematically investigate how the timing of cryotherapy affects sleep quality, including direct comparisons between morning and evening cryostimulation sessions.

No significant differences were observed between the PBC and control conditions in performance-related variables, except for pull-ups. Nevertheless, it is pertinent to contrast these findings with those elucidated in a previous study, where a series of ten PBC sessions yielded disparate outcomes relative to our study (Klimek et al., 2010). Specifically, this previous investigation conducted a series of 20-s Wingate tests both before and after the consecutive cryostimulation sessions, ultimately revealing a notable enhancement in anaerobic power among male participants following the intervention (Klimek et al., 2010). This incongruity in these outcomes appears to be attributable to variations in the duration of the intervention protocol. Notably, when cryotherapy is employed for post-exercise recovery purposes, substantiated positive outcomes were generally more evident with interventions extending over seven days or longer (Partridge et al., 2019; Rivera et al., 2018). The abbreviated duration of our study’s PBC protocol, spanning five days, may have contributed to the absence of significant effects observed in our evaluation.

In the evaluation of recovery outcomes, it is imperative to consider participants’ characteristics and the magnitude of training stress applied. Previous studies have shown that both local cryotherapy (foot cooling) and PBC using a cryocabin can enhance lower limb power performance (Hohenauer et al., 2020; Wu et al., 2023). Similarly, Fonda et al. (2013) observed that the application of PBC significantly accelerated the recovery of the rate of torque development in the hamstring muscle, highlighting cryotherapy’s potential restorative effects on jump performance. These findings appear to diverge from the outcomes derived from the present study. Nevertheless, it is imperative to underscore that our study introduced PBC in a ‘real-world’ setting, involving in-season elite athletes whereas the previous studies were conducted within a controlled laboratory setting with recreationally trained participants. The notable distinction in study settings prompts the assumption that participants in our study experienced more pronounced training stress. Specifically, participants in the present study engaged in three training sessions daily, accumulating approximately 5–6 h of training per day during the experimental period. In contrast, Hohenauer et al. (2020) applied five sets of plyometric exercises lasting approximately 20 min, and Fonda et al. (2013) incorporated five sets of plyometric exercises followed by six sets of eccentric hamstring exercises in their protocols. According to a previous study, when training stress does not exceed a certain threshold, it does not induce significant changes in HRV, an indicator of autonomic nervous system fatigue, or DOMS, a marker of local muscle fatigue (Medellín Ruiz et al., 2025). It is conceivable that PBC demonstrates its potential to enhance performance recovery under conditions of controlled training stress (e.g., laboratory-based training), while its efficacy becomes less certain when confronted with training stress exceeding the threshold.

Future research should investigate the effects of cryotherapy during alternate phases of the competitive season when training stress levels are comparatively diminished. Additionally, we suggest that future studies assessing performance-related variables in basketball players utilize a fatigue-inducing protocol, such as the one proposed by Vermeulen et al. (2024), and conduct performance tests under fatigued conditions. While the current study evaluated athletic performance in a non-fatigued state, basketball players spend 75% of their playing time at intensities exceeding 85% of their maximum heart rate (Stojanović et al., 2018) and must perform explosive movements under these conditions. Therefore, data collected under fatigued conditions are likely to provide a more accurate representation of the athletic performance required during real-game scenarios.

A noteworthy observation in the current study concerns the discernible decline in sleep quality exhibited by the study participants. Conventionally, the acceptable ranges for Wake After Sleep Onset (WASO) and sleep efficiency encompass values below 10% of total sleep duration and above 80% of total time in bed, respectively (Berger et al., 2005). In contrast, the WASO and sleep efficiency recorded for our subjects were approximately 26% of the total sleep duration and 79% of the total time in bed, respectively. Carter et al. (2020) reported that a relatively small proportion of athletes (6%) experienced sleep deprivation during the off-season. However, the authors hypothesized that sleep quality might deteriorate during the competitive season, especially in periods characterized by academic demands such as examinations (Carter et al., 2020). Remarkably, this hypothesis aligns with the outcomes of the current study, as the research was conducted within the context of the academic semester.

The present study has several strengths and limitations that warrant consideration. Notably, it is one of the few studies to investigate the impact of PBC on the sleep quality of elite athletes, an area that remains underexplored despite the growing popularity of cryotherapy in sports. Unlike prior research that predominantly focused on subjective sleep assessments, this study adopted a comprehensive approach by incorporating both subjective and objective sleep measures. Furthermore, the present study was conducted during the competitive season, a period characterized by elevated cumulative training stress. This setting reflects real-world conditions faced by athletes, enhancing the practical applicability of the findings.

One notable limitation of this study is the short exposure time of PBC. Participants in this study underwent PBC for an average of 128 s. As a result, a trend toward improved subjective sleep quality, as assessed by the PSQI-K, was observed in the PBC (−32.8%) compared to the control (−3.5%) condition. While this trend was supported by a large effect size (η_p_^2^ = 0.21), the improvement did not reach statistical significance. In contrast, previous studies employing longer exposure time of 180 s have shown significant improvements in sleep quality among physically active men and professional soccer players (Douzi et al., 2019a, 2019b). Ensuring meticulous control for cryotherapy exposure in future research may facilitate a more precise delineation of its impact on sleep quality. Nevertheless, it is important to acknowledge that certain individuals, particularly those characterized as cold-sensitive, may encounter challenges in completing the full 180-s cryotherapy protocol. To promote the widespread use of cryotherapy, it is essential to identify the minimum effective dosage that can prevent performance decline and sleep disturbances. The second limitation pertains to the exclusive use of the VAS to assess the intensity of muscle pain. In future studies, analyzing biochemical markers alongside the VAS would enable a deeper understanding of the effects of PBC on pain. Lastly, while the focus on in-season athletes provides valuable insights into the effects of PBC under conditions of high training stress, it also presents a limitation by restricting the applicability of the findings to other populations. The effects of PBC on sleep quality need to be examined across a wider range of contexts and with a larger and more diverse population.

## Conclusions

First, the present study demonstrated that PBC contributed solely to the recovery of a single athletic performance variable, i.e., the number of pull-ups performed. Second, five sessions of PBC were not able to improve subjective or objective sleep quality. However, the effect of PBC may vary depending on the magnitude of skin temperature reduction, and therefore the treatment dose should be set to achieve a decrease in skin temperature of approximately 15°C. Third, we observed poor sleep quality among our subjects. Collegiate athletes face not only sports-related stress, but also academic stress during the season, thus coaches and athletic trainers need to pay special attention to managing athletes’ sleep quality.
